# A healthy *Bifidobacterium dentium* caramel cocktail

**DOI:** 10.1016/j.jbc.2021.101452

**Published:** 2021-11-25

**Authors:** David Teze, Birte Svensson

**Affiliations:** 1 Enzyme Engineering and Structural Biology, The Novo Nordisk Foundation Center for Biosustainability, Technical University of Denmark, Kgs. Lyngby, Denmark; 2 Enzyme and Protein Chemistry, Department of Biotechnology and Biomedicine, Technical University of Denmark, Kgs. Lyngby, Denmark

**Keywords:** Glycoside hydrolase, GH172, caramel, prebiotic, difructo anhydride, enzyme mechanism, protein crystallography, molecular dynamics, CAZy, channel

## Abstract

β-d-fructofuranosyl glycosidases are enzymes that produce health-beneficial fructooligosaccharides from natural fructans. In a recent issue of JBC, Kashima *et al.* identified a novel α-d-fructofuranosyl-active enzyme, αFFase1, from the caries-associated bacterium *Bifidobacterium dentium*. αFFase1 reversibly forms a potential prebiotic also found in caramel, difructose dianhydride I, *via* intramolecular condensation of the substrate inulobiose. Kashima *et al.* elegantly combine NMR, X-ray crystallography, and molecular dynamics to describe an original mechanism for the reversible reactions catalyzed by αFFase1 that establishes the new glycoside hydrolase family GH172.

Knowledge of the diversity, digestive capacity, and metabolism of human gut microbiota is ever expanding, offering a treasure trove of novel enzymes that act on components present in our food. Fructose, one common such component, is naturally found in the fructan dietary fibers inulin and levan (containing β-(2->1) and β-(6->1) fructosylfructose linkages, respectively), well-known as commercial sources of prebiotic fructoligosaccharides that support the growth of healthy bacteria in the colon (1). Many related carbohydrate-active enzymes are described in the carbohydrate-active enzyme database (2), including well-characterized β-fructofuranosidases that act on the β-linkages in inulin and levan and that belong to glycoside hydrolase (GH) families 32 and 68. By contrast, the information is limited on enzymes that act on *α*-fructofuranosyl linkages, present in difructose dianhydrides (DFAs) known to be generated *via* the thermal treatment of fructose-containing foods, such as caramel (3, 4). Notably, caramel is quite rich in sugars with fructosyl-linkages such as inulobiose, DFA I, and diheterolevulosan II (4, 5). DFAs have been reported to possess beneficial health effects, such as improving the intestinal absorption of calcium and iron, lowering cholesterol, and stimulating the gut microbiota making the identification of enzymes that act on their *α*-fructofuranosyl linkages a significant topic.

Four DFA isomers have been shown to be produced by microbial strains that degrade fructans (3). For example, members of the GH91 family of inulin fructotransferases (2, 3, 6), which have a right-handed parallel β-helix structural fold catalyze the formation of DFAs I (α-d-Fru*f*-1,2’:2,1′-d-β-Fru*f*) and III (α-d-Fru*f*-1,2’:2,3′-d-β-Fru*f*) from inulin *via* a single-step inverting mechanism and the formation of an α-from a β-fructosyl-linkage (6). Several *Bifidobacteria* species from human gut microbiota have been shown to grow on α-d-Fru*f*-(2->6)-d-Glc obtained from d-glucose and d-fructose by thermal treatment (5), suggesting that these species contain an α-fructofuranosidase. Therefore, Kashima *et al.* (7) set out to discover the gene encoding this fructose-metabolizing enzyme in a strain of *Bifidobacterium dentium* isolated from human dental caries that also resides in the human gut and can grow on DFA I as a sole carbon source (7, 8). They identified an operon of genes that encoded a Lac1-family transcriptional regulator, a putative GH32 β-d-fructofuranosidase, a hypothetical protein (locus tag BBDE 2040) belonging to a domain of unknown function family 2961, and a set of putative ABC transporter proteins (7). Upon closer investigation of the BBDE 2040 gene product, the recombinant protein produced in *Escherichia coli* was found to act on α-fructosyl linkages in caramelized sugar and was thus named difructose-dianhydride synthase/hydrolase (αFFase1) (7). αFFase1 was shown to be able to catalyze intramolecular dehydration (condensation) of inulobiose (β-d-Fru*f*(2->1)-d-Fru) *via* the formation of α-linkages in DFA I and diheterolevulosan II (α-d-Fru*f*-1,2':2,1′-β-d-Fru*p*) and the corresponding reverse reactions (7) (Fig. 1*A*).

**Figure 1:**
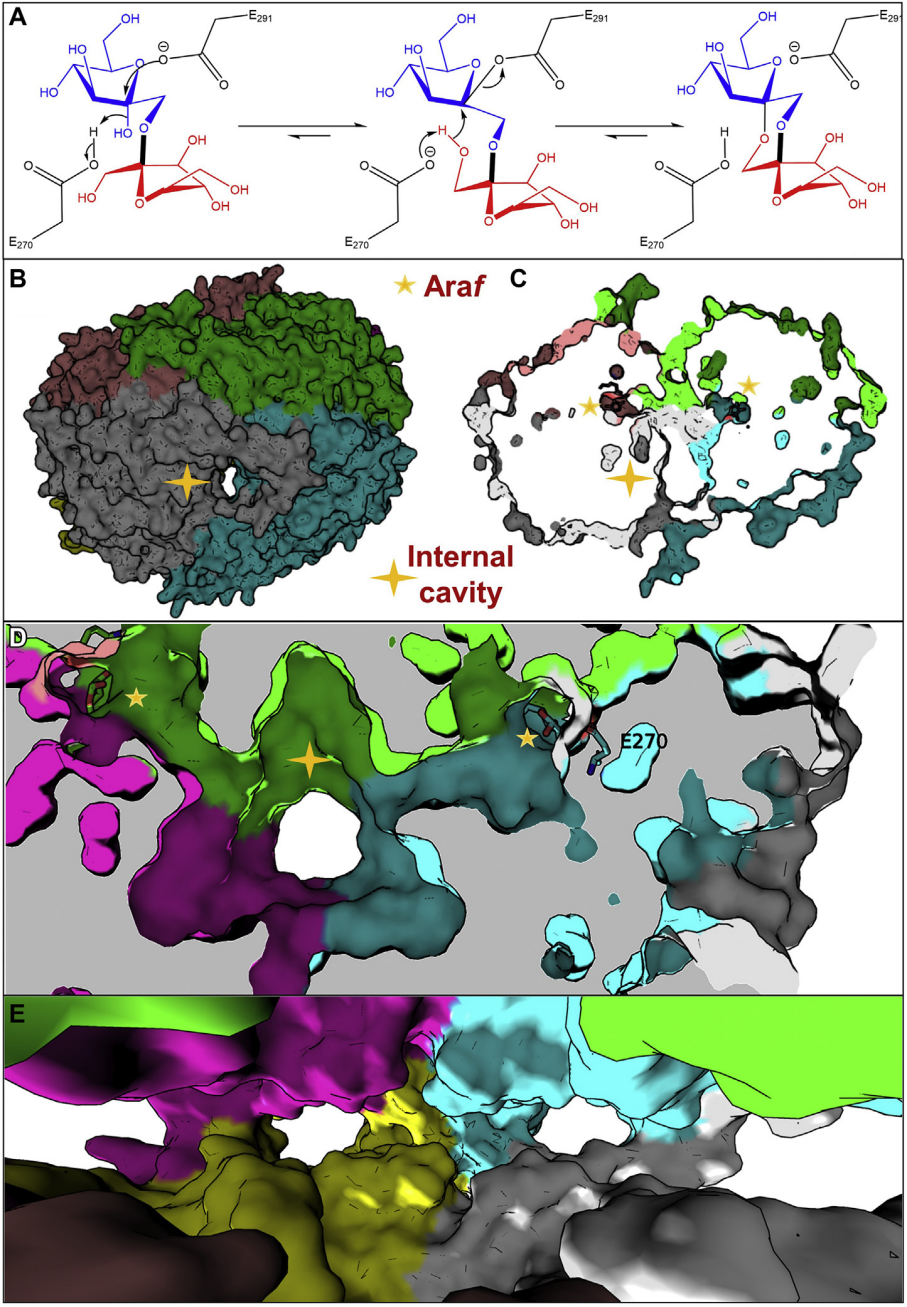
**αFFase1 fold and mechanism.***A*, mechanism of condensation of inulose to form DFA I catalyzed by αFFase1. In the first step, type 2 nucleophilic substitution, E291 attacks the C-2 of the reducing (*blue*) sugar of inulose, forming a β-fructosyl linkage, whereas the anomeric hydroxyl departs as a water molecule, abstracting a proton from E270. In the second step, the OH-1 of the second fructosyl residue (*red*) is deprotonated by E270 and attacks the anomeric C-2 of the first sugar (*blue*), cleaving the glycosyl-enzyme bond. *B*‒*E*, surface representations of the αFFase1 hexamer (PDB ID 7V1W) with each protomer colored differently. The four-pointed stars indicate the internal cavity, whereas the five-pointed stars indicate bound Ara*f* and thus the active sites. *B*, external view of the protein surface. *C* and *D*, slice views of the interior, with the active sites linked through the channels to the internal cavity. *E*, view from the internal cavity, showing connections from the cavity to the exterior (*bulk solvent*). αFFase1, α-d-fructofuranosidase 1; DFA, difructose dianhydride.

To elucidate the catalytic mechanism of αFFase1, the authors first used ^1^H NMR to assess the stereochemical outcome of the reaction on a model substrate (*p*-nitrophenyl α-d-arabinofuranoside, *p*NP-α-d-Ara*f*) (7). Based on this, *a*FFase1 was determined to be an α-furanosidase that acts *via* a retaining mechanism, forming an α-fructosyl bond from another α-fructosyl bond through two successive type 2 nucleophilic substitutions (Fig. 1*A*), in contrast to the inverting mechanism of family GH91 enzymes. The high resolution crystal structures of ligand-free, fructofuranose-, and arabinofuranose-complexed αFFase1 showed a hexamer with a large internal cavity connected through six channels (that formed at the interface between protomers) to the active sites (Fig. 1, *B*–*E*). These active sites had no direct connection to solvent except through the channels and internal cavity, which itself has connections to the exterior (Fig. 1*E*). The overall fold of each protomer consisting of two β-jelly roll domains and an α-helix was previously unreported for a GH enzyme. Two catalytic residue candidates (E270 and E291) were identified in the ligand:*a*FFase1 complex, and their roles were confirmed by mutagenesis and kinetic studies. Long (1 μs) molecular dynamics (MD) simulations were performed of the full hexamer complexed with either fructose, DFA I, or inulobiose at all six active sites. Most of the sugar molecules stayed in the active sites during the whole simulation, though a few reached the internal cavity through the channels, displaying unusual channeling of substrates and products.

Because the two most common forms of retaining mechanisms of GH enzymes were first established, only a handful of others have been described (9, 10). Therefore, novel retaining mechanisms are rare within the GH community. Although *a*FFase1 hydrolyzes *p*NP-α-d-Ara*f via* the classic retaining mechanism, it catalyzes the condensation of inulobiose through an original mechanism (7) previously unseen. The first step of the condensation reaction (favored over hydration) resembles that of reverse hydrolysis by classic-retaining GHs, with the formation of a glycosyl-enzyme intermediate and departure of a water molecule (formerly the anomeric OH-2 of fructose in subsite −1), mediated by the catalytic acid-base residue E291 (Fig. 1*A*). This positioning of the reducing residue in subsite −1 and the nonreducing residue occupying subsite +1 is noteworthy, as it is contradictory to the definition of subsites in GHs. On the other hand, the subsequent deglycosylation step is more akin to the inverting mechanism of the DFA-forming GH91 (6) or to the mechanism of lytic transglycosylases (9), with an intramolecular nucleophilic attack forming a dioxane cycle that links the two anomeric carbons in the tricyclic product (7).

The αFFase1 crystal structures and biochemical studies of Kashima *et al.* (7) documented a hitherto unreported reaction mechanism, and prediction of gut bacterial orthologues of αFFase1 suggests that its evolution stems from the time period of ancient bread baking. These findings add to our understanding of yet another facet of the wide nutrient space metabolized by bacterial symbionts in the digestive tract of modern humans. The detailed characterization of αFFase1 (7) provides an excellent starting point for further studies on how various fructose-derived oligosaccharides such as inulobiose and tricyclic anhydrides such as DFA I are metabolized to form health-beneficial compounds by various *Bifidobacteria* encoding αFFase1 orthologues (7). Moreover, in the light of commercial interests in functional food additives of fructan-/fructose-origin (1, 3) and the health benefits of fructose-derived caramel, *a*FFase1 may offer a route to novel enzyme-catalyzed scalable industrial processes.

## Conflict of interest

The authors declare that they have no conflicts of interest with the content of this article.
